# Monitoring of Cardiorespiratory Parameters in Rats—Validation Based on Pharmacological Stimulation

**DOI:** 10.3390/ph14121223

**Published:** 2021-11-25

**Authors:** Joanna Miklosz, Bartlomiej Kalaska, Stanislaw Zajaczkowski, Dariusz Pawlak, Andrzej Mogielnicki

**Affiliations:** 1 Department of Pharmacodynamics, Medical University of Bialystok, 15-089 Bialystok, Poland; bartlomiej.kalaska@umb.edu.pl (B.K.); dariusz.pawlak@umb.edu.pl (D.P.); andrzej.mogielnicki@umb.edu.pl (A.M.); 2 Department of Physiology, Medical University of Gdansk, 80-211 Gdansk, Poland; stanislaw.zajaczkowski@gumed.edu.pl

**Keywords:** adrenaline, drug development, methods, nitroglycerin, rat, safety pharmacology, toxicology, translation

## Abstract

The methods used in preclinical studies should minimize the suffering and the number of animals but still provide precise and consistent results enabling the introduction of drug candidates into the phase of clinical trials. Thus, we aimed to develop a method allowing us to perform preliminary safety and toxicity studies of candidates for human medicines, while reducing the number of animals. We have devised a method based on a combination of two devices: Plugsys (Transonics System Inc., Ithaca, NY, USA) and PhysioSuite (Kent Scientific Corporation, Torrington, CT, USA), which allow simultaneous registration of nine circulatory and respiratory parameters, and body temperature. Vehicle and adrenaline, or nitroglycerin, as reference substances were administered into the right femoral vein of Wistar rats. Physiological conditions were registered over 60 min after drug administration by measuring systolic, diastolic and mean blood pressure, heart rate (HR), blood perfusion of paw vessels, blood oxygen saturation, respiratory rate, average and peak exhaled CO_2_, and body temperature. Blood pressure was measured by cannula placed in the left common carotid artery and connected to the pressure transducer (Plugsys). The other parameters were measured by the PhysioSuite. Adrenaline-induced immediate dose-related hypertension and nitroglycerin hypotension were correlated with the change in blood perfusion. They both increased HR. Adrenaline decreased blood oxygen saturation and slightly affected respiratory parameters, while nitroglycerin caused a progressive increase in respiratory rate and a decrease in the peak of exhaled CO_2_. Our method may become an inseparable part of the preliminary safety and toxicity studies of tested drugs, while being an important step towards improving animal welfare.

## 1. Introduction

Validated animal models are required for the approval of testing drug candidates in humans and can also deliver valuable translatable endpoints for predicting safety. Food and Drug Administration recommends testing the effects of pharmaceutical compounds on vital functions in laboratory animals prior to first-in-human studies. The cardiovascular, respiratory, and central nervous systems are thought to be the vital organ systems that should be examined in a “core battery” of safety pharmacology assays. Substances which are considered to be cardiotoxic in animals are usually cardiotoxic in humans, thus their preclinical evaluation is particularly important [1,2,3]. Unchanged systolic, diastolic and mean blood pressure (BP), heart rate (HR), electrocardiogram, human ether-a-go-go-related gene potassium channels, blood oxygen saturation, respiratory rate, tidal volume and optionally airway resistance, pulmonary arterial pressure, blood pH, and blood gases guarantee the passage of the experimental drugs into the next stage of research. European Medicines Agency expect that two species—a rodent and non-rodent—will be used to test compounds by the intended clinical route, for a duration of at least the length of the proposed clinical trial, at multiple doses with the highest dose exceeding the proposed clinical dose. Rats are useful in drug research for assessing the effects of novel compounds on cardiovascular and respiratory function because of their well-known anatomy, physiology, and metabolism [4,5,6,7,8,9]. The rat model provides the added advantages of lower costs and compound requirements. With such a wide application of the model and its significance in early decision-making, it is important to understand the implication of effects observed on cardiorespiratory parameters and their potential translation to other preclinical animal studies prior to human trials.

We developed a new method which facilitates the monitoring of all main cardiovascular and respiratory parameters in one rat at the same time. Our design configuration helps to reduce the number of animals in the preclinical studies as test candidates for human pharmaceuticals. The method combines both direct and indirect techniques and consists of two devices: Plugsys (Transonics System Inc., Ithaca, NY, USA) and PhysioSuite (Kent Scientific Corporation, Torrington, CT, USA). The aim was to determine whether our method is reliable and can be used for further research. As the pharmacological mechanism of action of adrenaline and nitroglycerin on both animal and human subjects is well researched and understood, these compounds were selected for the study in order to facilitate development of a standardized cardiorespiratory monitoring protocol.

## 2. Results

### 2.1. Blood Pressure

**Figure 1:**
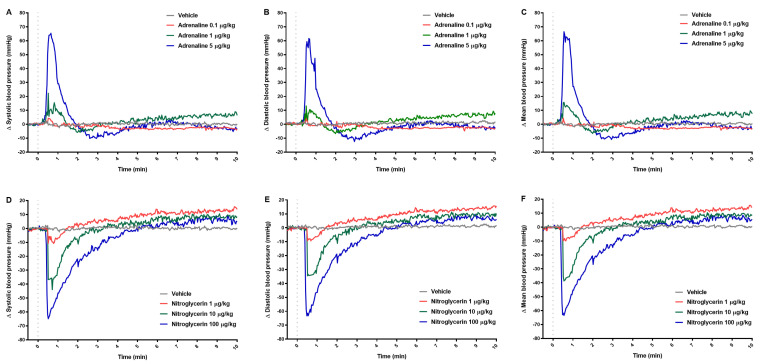
The effect of adrenaline (**A**–**C**) and nitroglycerin (**D**–**F**) on systolic, diastolic, and mean blood pressure. The course of blood pressure registered for 10 min after intravenous injection (gray dotted line) of vehicle or tested drugs. Results are shown as median (line). n = 5 in the group treated with tested drugs and n = 8 in vehicle group.

Both tested compounds changed BP in a dose-dependent manner. The course of changes in systolic, diastolic, and mean BP was similar within the studied group (Figure 1A–F). Adrenaline at the lowest dose did not change BP significantly. The injection of adrenaline at medium dose increased BP rapidly within the first minute, maximally by 17% (122.0 ± 21.2 vs. 142.2 ± 21.0), 12% (117.1 ± 20.0 vs. 131.2 ± 16.1), and 15% (119.3 ± 20.6 vs. 137.2 ± 18.1), for systolic, diastolic, and mean BP, respectively. Similarly, adrenaline at the highest dose increased BP, maximally by 49% (118.1 ± 9.5 vs. 176.4 ± 13.7), 46% (113.1 ± 9.0 vs. 165.5 ± 15.5), and 47% (115.9 ± 9.4 vs. 170.7 ± 14.4), for systolic, diastolic, and mean BP, respectively. Just after the increase of BP, we observed a temporary decrease in BP lasting a few minutes when adrenaline was administered at the highest dose (Table 1 and Table S1).

The nitroglycerin administered at a dose of 10 µg/kg significantly decreased systolic (127.4 ± 14.2 vs. 89.3 ± 19.2), diastolic (120.5 ± 13.7 vs. 84.5 ± 18.1), and mean BP (124.1 ± 14.1 vs. 87.0 ± 18.5) just after starting of injection, maximally by 30% in all groups. Similarly, nitroglycerin at a dose of 100 µg/kg decreased BP, maximally by 54% (118.8 ± 6.1 vs. 54.2 ± 9.9), 55% (114.7 ± 5.1 vs. 51.4 ± 8.7), and 55% (116.8 ± 5.6 vs. 52.9 ± 9.5), for systolic, diastolic, and mean BP, respectively. After the rapid decline, there was a gradual slight increase in BP which was statistically significant when nitroglycerin was administrated at the lowest dose (Table 1 and Table S1).

### 2.2. Blood Perfusion of Paw Vessels, Heart Rate, and Blood Oxygen Saturation

**Figure 2:**
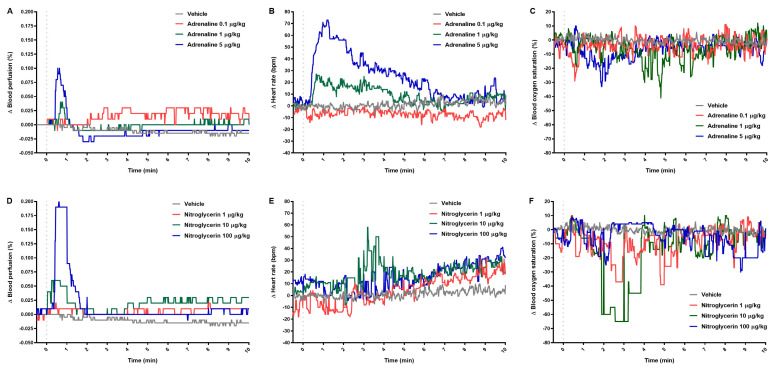
The effect of adrenaline (**A**–**C**) and nitroglycerin (**D**–**F**) on blood perfusion of paw vessels, heart rate, and blood oxygen saturation. The course of tested parameters registered for 10 min after intravenous injection (gray dotted line) of vehicle or tested drugs. Results are shown as median (line). n = 5 in the group treated with tested drugs and n = 8 in vehicle group.

Adrenaline produced a dose-dependent effect on the test parameters (Figure 2A–C). When administered at the highest dose, it increased blood perfusion of paw vessels and HR maximally by 100% and 21%, respectively (Table 2). Adrenaline injected at the highest dose decreased blood oxygen saturation within the first minutes and after a half hour, maximally by 27% (Table 2). We observed a clear dose-dependent effect on blood perfusion of paw vessels reaching maximally 100% and 400%, when nitroglycerin was administered at middle and at the highest dose, respectively (Table 2). After immediate increase, blood perfusion of paw vessels returned to normal level and then gradually increased (Figure 2D). Nitroglycerin both at the middle and at the highest dose affected HR significantly, and blood oxygen saturation at the lowest dose, but these changes were weaker (below 10%) than in the adrenaline-treated rats (Figure 2E,F; Table 2).

**Figure 3:**
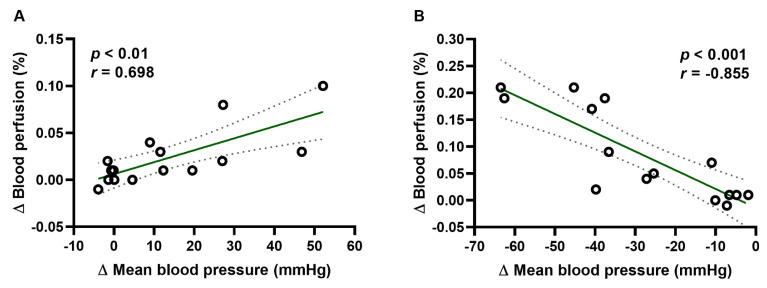
Correlation between change of blood perfusion of paw vessels and change of mean blood pressure for rats treated with adrenaline (**A**) and nitroglycerin (**B**) at the first minute of the experiment. Each data point represents a blood perfusion of paw vessels value corresponding to a mean blood pressure value. The line of best fit (solid line) and 95% confidence limits (dashed lines) for all data points are indicated.

The significant positive (r = 0.698; *p* < 0.01; Figure 3A) and negative (r = −0.895; *p* < 0.001; Figure 3B) correlation was observed between blood perfusion of paw vessels and mean BP in rats treated with adrenaline and nitroglycerin, respectively. 

### 2.3. Respiratory Parameters

**Figure 4:**
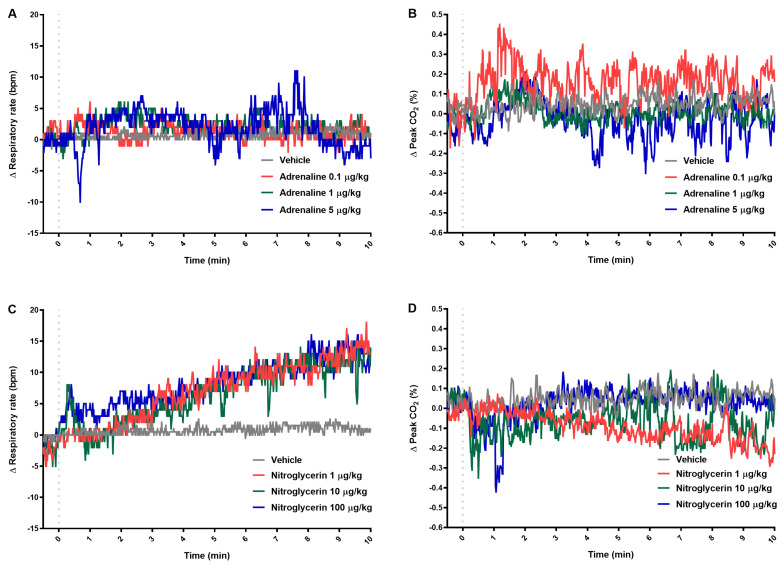
The effect of adrenaline (**A**,**B**) and nitroglycerin (**C**,**D**) on respiratory rate and peak CO_2_. The course of tested parameters registered for 10 min after intravenous injection (gray dotted line) of vehicle or tested drugs. Results are shown as median (line). n = 5 in the group treated with tested drugs and n = 8 in vehicle group.

Adrenaline did not affect measured respiratory parameters although some trends could be observed (Figure 4A,B). When administered at low and at the highest dose, it slightly increased the respiratory rate in the first minutes, and at the middle dose, decreased the peak of exhaled CO_2_ after a half-hour (Table 3). Nitroglycerin injected at all doses caused a progressive increase in respiratory rate and decrease in the peak of exhaled CO_2_ (Figure 4C,D; Table 3).

### 2.4. Results Obtained by Separated Methods

**Figure 5:**
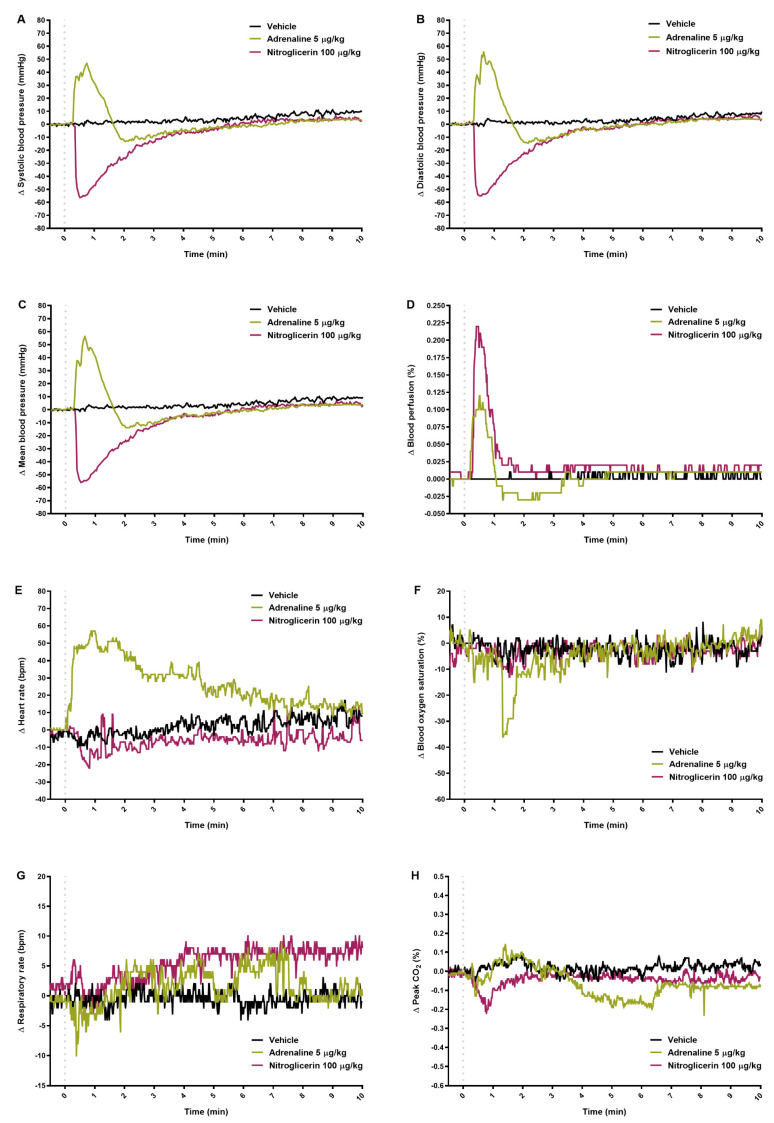
The effect of adrenaline and nitroglycerin on systolic (**A**), diastolic (**B**), and mean blood pressure (**C**) measured by Plugsys (Transonics System Inc.) separately, and on blood perfusion of paw vessels (**D**), heart rate (**E**) and blood oxygen saturation (**F**), respiratory rate (**G**), and peak CO_2_ (**H**) measured by the PhysioSuite Physiological Monitoring Modular System (Kent Scientific Corporation), separately. The course of parameters registered for 10 min after intravenous injection (gray dotted line) of vehicle or tested drugs. Results are shown as median (line). n = 5.

Within the first minute, adrenaline and nitroglycerin at the highest doses significantly increased or decreased systolic, diastolic, and mean BP, respectively (Figure 5A–C; Table S2). Both drugs affected blood perfusion significantly (Figure 5D; Table S3). Adrenaline increased HR and decreased blood oxygen saturation just after starting of injection (Figure 5E,F; Table S3). Adrenaline did not affect measured respiratory parameters while nitroglycerin caused decrease in the peak of exhaled CO_2_ (Figure 5G,H; Table S4).

## 3. Discussion

We combined direct and indirect methods to monitor cardiovascular and respiratory parameters at the same time in the same animal. This way we limited the number of animals, while still replicating previous separate experiments measuring cardiovascular and respiratory parameters. As expected, we observed changes in systolic, diastolic, and mean BP, HR, blood oxygen saturation, blood perfusion of paw vessels, peak exhaled CO_2_, and respiratory rate after a single intravenous injection of natural hormone, classic sympathomimetic agent—adrenaline and nitric oxide-generating drug—nitroglycerin. We proved our experimental setup may be successfully used to test acute toxicity of drug candidates administered by intravenous single administration. Although the model requires anesthesia, it does not need long-term acclimatization of animals, and it could therefore help in quick identification and elimination of chemical compounds affecting cardiovascular or respiratory systems at the outset of the drug discovery process.

The draft guideline of the International Conference on Harmonization of Technical Requirements for Registration of Pharmaceuticals for Human Use (ICH) S7B (2004) considered an anesthetized model as valuable despite the fact that ICH S7A guideline (2001) recommends the use of conscious animals. The optimal anesthesia must be selected in accordance with the study objective and should minimally affect animal physiology and maximally eliminate their stress and suffering. Anesthetized animals may have lower body temperature than awake animals. Additional care must be taken to maintain the proper body temperature, as hypothermia depresses respiration and cardiac functions [10]. In the present study, rats were placed on a warming pad. PhysioSuite accurately monitored the body temperature and controlled pad temperature to warm the animal with two temperature sensors, for the animal and the pad. Rats were anesthetized with an intraperitoneal injection of sodium pentobarbital—a commonly used barbiturate in laboratory practice, with known dose- and species-dependent effect on the circulatory and respiratory system [11,12]. The barbiturates interfere with Ca^2+^ transport in cardiac myocytes and with Ca^2+^ uptake into the sarcoplasmic reticulum and it has a dose-dependent negative inotropic effect. They may also interfere with Ca^2+^-mediated events and generally tend to result in an increased preload, decreased afterload, and increased HR. Barbiturate-induced tachycardia is attributed to a depression of baroreceptor reflex afferent mechanisms. This results in a lack of inhibition of constrictor tone and depression of vagal and sympathetic components of the baroreceptor–HR reflex. When compared to measurements made in conscious rats, pentobarbital lowered ejection fraction, fractional shortening, fractional area change, and velocity of circumferential fiber shortening, but these effects were less pronounced than after isoflurane or ketamine/xylazine [13]. According to the literature, the dose of 35–40 mg/kg minimally changes the BP, HR, and cardiac output in rats [14,15]. Pelaez et al. showed that BP measurements obtained by direct method from rats anesthetized with thiobutabarbital are strongly correlated with the values received by telemetry in awake animals [16]. It has also been shown that hemodynamic, echocardiographic, and biochemical parameters were largely unaffected by barbiturates in rats. BP remained constant and was similar in the conscious rats and pentobarbital groups [17,18]. As we expected, we did not observe any significant effect of sodium pentobarbital on the cardiovascular and respiratory parameters in the control group.

Our method uses direct (invasive) techniques for measuring BP, respiratory rate, average and peak CO_2_, and indirect (noninvasive) technique for measuring HR, blood oxygen saturation, and blood perfusion of paw vessels. Direct measurements of BP, such as the external catheter system used in our study and telemetry, are preferable in comparison to indirect techniques because of their ability to accurately, reliably, and comprehensively monitor the dynamic nature of this parameter. Drug candidate effect on BP evaluated by the indirect method still raise questions and should be taken with caution. The indirect methods are useful for screening substantial changes of BP among large number of animals [10,17]. Many indirect methods are faced with some challenges such as the difficulty of continuous recording or exceeding thresholds over the course of experiment [19]; thus, these techniques are not recommended to quantify the relationship between BP and other variables [17]. In experimental medicine, telemetry has become a gold standard for BP, HR, electrocardiogram, and body temperature monitoring because of its miniaturization and ability for direct high-fidelity recording over days, weeks, and even months without the need for restraint or chemical-induced sedation in free-moving animals of different species [17,20,21,22]. The conscious telemetered rat is widely used as an early in vivo screening model for assessing the cardiovascular safety of novel pharmacological agents administered extravascularly. The consistency of the results and decreased interanimal variability reduce the number of animals needed for single study [20]. Nevertheless, our method can provide nearly all of the same advantages as the telemetry techniques [17]. However, in telemetry one type of gauge is connected with the whole wireless system and animals have to be isolated in individual cages for transmitter signals [23]. It is also not possible to check system calibration during the experiment [17]. Telemetry BP measurement is successfully used to evaluate long-acting drug candidates administered extravascularly and chronically, while the procedure we have developed is for the assessment of short-acting compounds administered intravenously, in life-threatening situations. Both methods require advanced surgical skills. Telemetry involves implantation of transmitters while the method we present requires fluid-filled catheters externally connected to the artery [23]. The catheter was placed in the carotid artery and connected to the calibrated pressure transducer and recording device. The failure of arterial catheterization in extreme cases can lead to inflammation or thrombosis with necrosis of the distal tissues [24]. In the telemetry model, long-term presence of the device itself, might cause significant adverse physiological effects, stress, and discomfort [25]. In chronic experiments, the initial implantation surgery required under anesthesia frequently results in the animals needing about week to recover after awakening. Unwanted infections or surgical wound complications may occur [26]. Despite the efforts to acclimatize animals undergoing indirect procedures, these methods also impose significant stress that disturbs multiple aspects of the cardiovascular system such as significant increases in BP and HR in rodents [27,28]. Although it has been recommended to train animals for 7 to 14 days before commencing tail-cuff measurements, some investigators have demonstrated that even 10 days failed to prevent the large changes in BP and HR induced by stress [27,28]. We chose the direct techniques for measuring BP and respiratory parameters because, like telemetry, they were proven to be precise, and allow for continuous measurements, but unlike telemetry, they are cheaper and less time-consuming. Our method does not require such long acclimatization. The results of the control group show no stress effect on the cardiorespiratory parameters. Moreover, appropriate anesthesia limits to the greatest extent the suffering of the individual animal which is reduced to only to the initial puncture. 

The advances in direct measurement technology are shifting attention away from indirect techniques, but these methods still provide an advantageous approach in some experimental circumstances. We measured BP and respiratory parameters directly, but in order to minimize the invasiveness of the whole method and increase the number of tested parameters at the same time, we applied additional indirect techniques. Delaunois et al. showed that a system combining noninvasive plethysmography and invasive telemetry allows simultaneous monitoring of parameters with the appropriate sensitivity and data is similar to those obtained from separate methods [29]. Photoplethysmography is a light-based technology using a light source to record the pulse signal wave. It measures the intensity of light fluctuations penetrating tissue which reflects the change of blood perfusion of paw vessels. The light from the working environment, animal movements, and skin pigmentation can affect the results, which are poorly correlated with the results of direct methods. Therefore, photopletysmography is the least recommended for BP measurement in rodents [10]. In our study HR, blood oxygen saturation and blood perfusion of paw vessels were determined by passing two wavelengths of low intensity light, one red and one infrared, through the body tissue to a photodetector. We chose the pulse oximeter placed on the paw because it is user-friendly and does not interfere with direct measurement of BP and respiratory parameters. We obtained a wide range of cardiovascular parameters and limited the number of animals. The use of anesthesia and albino rats additionally increased the accuracy of the results obtained by photopletysmography. We found that blood perfusion of paw vessels and mean BP are strongly correlated with each other in the nitroglycerin and adrenaline treated groups. It seems that noninvasive blood perfusion measurement could be a good predictor of the effect of tested compounds on BP. Thus, it would allow us to limit invasive BP measurement and additionally minimize animal suffering. We showed similar effects of adrenaline and nitroglycerin in the experiment with both devices used separately or combined, indicating that both procedures do not interfere with each other. However, thanks to our modified method, we are able to reduce the time of experiment and the number of animals by half while providing reliable data. It is currently impossible to avoid using animals during testing new candidates for human medicines, although our method clearly addresses the 3R rule.

According to the ICH S7A guidance, respiratory rate and other measures of pulmonary function, such as tidal and minute volume, should be evaluated and quantified. The methods based on plethysmography are commonly used to establish the effects of drug candidates on respiratory function. Some laboratories perform such testing in rodents using a whole-body technique and others using a head-out procedure [30]. Both techniques measure chamber pressure to determine changes in ventilatory function, which most often includes the parameters of respiratory rate, tidal and minute volume, but can also include other outputs such as inspiratory/expiratory time and peak inspiratory/expiratory flow. With the whole-body plethysmography method, the animals are free to move around the test chamber, and the alterations in the pressure in the chamber due to the exhaled air having been warmed and humidified in the animals’ lungs are quantified. With the head-out method, the animals are restrained due to a sealed neck collar, and the changes in the pressure in chamber due to thoracic cavity movement are measured. All of the above techniques preclude the simultaneous measurement of cardiovascular parameters. In our method, we performed a tracheotomy in rats and put the sensor in the tracheal tube connected to a capnometer which used infrared absorption spectroscopy measured respiratory rate, average, and peak exhausted CO_2_. Due to the dynamic nature, average exhausted CO_2_ is not a parameter for simple quantification of respiratory function. Because of its stable nature, peak exhausted CO_2_ is much better suited for this role. We cannot examine other respiratory parameters, but the ones we measure are sufficient to assess general effects of compounds on the respiratory system. Again, we limited the number of animals in a single study because we measure both cardiovascular and respiratory parameters simultaneously.

The nonclinical safety studies should be designed to characterize the toxic effects and toxicity target organs, and to define the dose-dependent relationship of the adverse effect. If it is possible, the time course of any side effects should be investigated. The doses should include and exceed the therapeutic range to compare toxic doses with doses producing the pharmacodynamic effect [31]. In our method, we are able to indicate qualitatively whether the target of toxicity is the cardiovascular or respiratory system, and to quantify the safe exposure level. Our method allows us to estimate the no-observed-adverse-effect level and the maximum tolerated dose. We chose different chemical classes of compounds to assess the ability of the model in differentiating the effects driven by distinct pharmacological mechanisms [30]. Adrenaline with positive inotropic and chronotropic effects is commonly injected intravenously for some life-threatening conditions such as cardiovascular collapse, asystole, ventricular fibrillation, or anaphylactic shock [32]. We have investigated well-known effects of this stimulator of α- and β-adrenergic receptors on cardiorespiratory parameters in rats using our method. Cutaneous blood vessels express almost exclusively α receptors while the smooth muscle of blood vessels supplying skeletal muscles express both β_2_ and α receptors. Adrenaline at physiological concentrations has greater influence on β_2_-vasodilator receptors, but at high concentrations, the response from α receptors predominates [33]. Stimulation of vascular α_1_-receptors causes an immediate vasoconstriction and thus an increase of BP followed by a subsequent decline due to stimulation of vascular β_2_-receptors [32,33,34,35]. Adrenaline, as a potent bronchodilator, relaxes bronchial muscle, making breathing easier and faster [33]. The increase of respiratory rate was observed immediately after the injection. Effects on BP and HR are related to plasma adrenaline concentration, which is also influenced by the route of administration [32]. We injected adrenaline intravenously at doses commonly found in the literature [32,34]. The rapid dose-response increase in systemic arterial BP followed by a decline occurred simultaneously with increased perfusion of paw vessels, which was correlated with each other. Adrenaline also in a dose-dependent manner, produced an immediate increase in HR and a decrease in blood oxygen saturation, followed by a slow return of parameters to the starting level. The change in blood oxygen saturation can reflect its vasoconstrictive effect which disturbs measurement of blood saturation or can be caused by adrenaline calorigenic action which is known to increase oxygen consumption [33,36].

The second reference drug was nitroglycerin, one of the most known nitric oxide donors used clinically in hypertensive emergencies or to dilate coronary blood vessels. In our study, intravenously administered nitroglycerin induced an immediate and dose-dependent reduction in BP which returned rapidly to normal values as expected [37]. Nitric oxide relaxes directly vascular smooth muscle cells via activation of guanylate cyclase, increasing intracellular levels of cyclic guanosine monophosphate, resulting in activation of protein kinase G, which is responsible for the phosphorylation of proteins involved in vasorelaxation [38]. The hypotension and increased perfusion of the paw vessels demonstrates the vasorelaxant effect of nitroglycerin. These two effects strongly correlated with each other. We also observed a slight increase in HR. The tachycardic response produced by nitroglycerin is the most likely baroreflex-mediated [39]. Perhaps in our method, the baroreceptor reflex was inhibited by the pentobarbital used to anesthetize rats [32]. Nitric oxide donors at extremely high doses change respiration biphasically, with initial bradypnea followed by a tachypnea [39]. In our study, we did not notice a significant decrease in respiratory rate, but only a progressive increase accompanied by a decrease in peak exhaled CO_2_. The above findings are consistent with known effects of nitric oxide donors and adrenergic agonists which confirms the accuracy and sensitivity of our method.

## 4. Materials and Methods

### 4.1. Ethics Statement

Studies were performed in accordance with European Parliament and Council Directive 2010/63/EU on the protection of animals used for scientific purposes, Animal Research: Reporting of In Vivo Experiments guidelines and national regulations. All procedures involving animals were accepted by the Local Ethics Committee (permit numbers: 108/2015; 2/2018).

### 4.2. Drugs

Adrenaline was purchased from Sigma Aldrich (Darmstadt, Germany), nitroglycerin from Merus Labs (Amsterdam, Nederland), and unfractionated heparin from Polfa Warszawa (Warsaw, Poland). Pentobarbital sodium was obtained from Biovet (Pulawy, Poland), phosphate buffered saline (PBS) from Biomed Lublin (Lublin, Poland) and sodium chloride solution 0.9% from Baxter (Warsaw, Poland).

### 4.3. Animals

Male Wistar rats were obtained from the Centre for Experimental Medicine at the Medical University of Bialystok. The animals were bred in a 12-h light/dark cycle in a temperature and humidity controlled room. The animals were grouped into cages as appropriate and allowed to have ad libitum access to sterilized tap water and standard chow in specific pathogen-free conditions. After the end of the experiment, all animals were euthanized by cutting the heart muscle and exsanguination.

### 4.4. Experimental Procedure Performed with Both Methods Simultaneously

**Figure 6:**
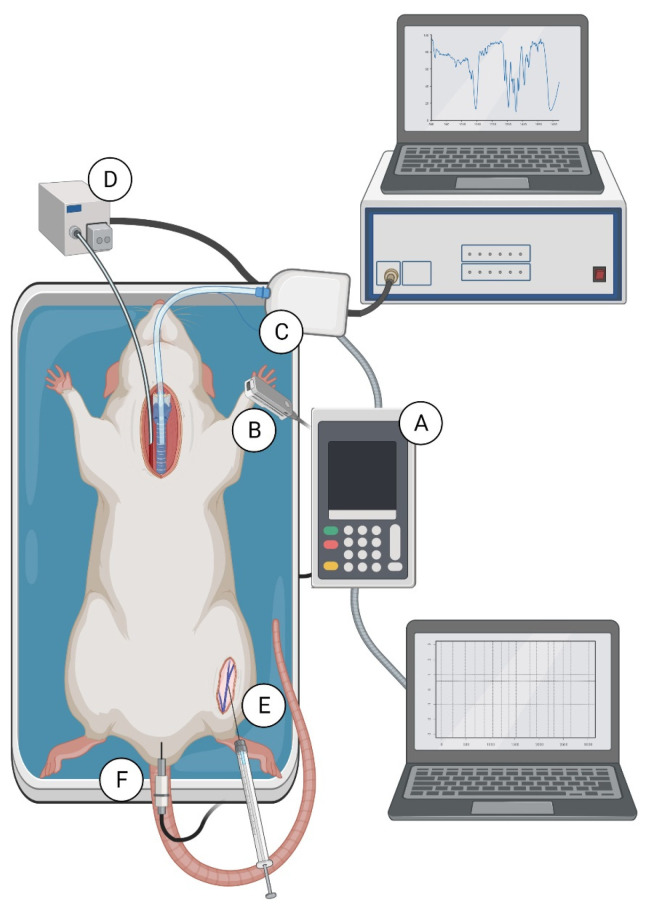
Monitoring of vital parameters on rat. PhysioSuite Physiological Monitoring Modular System (**A**) connected with pulse oximeter—MouseSTAT module (**B**) and respiratory rate sensor—CapnoScan modul (**C**). Catheterization of right carotid artery for blood pressure measurement with the pressure transducer (Plugsys, Transonics System Inc.) (**D**). Catheterization of right femoral vein for drug administration (**E**). Placement of temperature sensor (**F**).

Thirty-eight male Wistar rats (12–15 weeks old, weighing 238.9 ± 26.8 g) were randomly divided into 7 groups, anesthetized by an intraperitoneal injection of pentobarbital (45 mg/kg), and placed in a supine position on a heated operation table. Supplemental pentobarbital (5 mg/kg) was administered as needed, with a delay of at least 10 min before any experimental measurements were made. HR, blood perfusion of rat’s paw, blood oxygen saturation, respiratory rate, average and peak exhaled CO_2_, and body temperature were measured using the PhysioSuite Physiological Monitoring Modular System (Kent Scientific Corporation, Torrington, CT, USA) for 60 min after drugs administration (Figure 6A). The MouseSTAT module recorded HR, blood perfusion of rat’s paw and blood oxygen saturation using a rat paw pulse oximeter (Figure 6B). The CapnoScan module measured respiratory rate, average and peak exhaled CO_2_ using a sensor placed in the tracheal tube (Figure 6C). Systolic, diastolic, and mean BP were measured directly through a cannula filled with unfractionated heparin solution (150 U/mL), placed in the left common carotid artery and connected to the pressure transducer (Plugsys, Transonics System Inc., Ithaca, NY, USA) (Figure 6D). The drugs were administered after the stabilization of measured parameters at doses selected based on the literature [32,34,37]. Adrenaline (0.1, 1 and 5 µg/kg, 1 mL/kg) and nitroglycerin (1, 10 and 100 µg/kg, 1 mL/kg) were administered into the right femoral vein. Vehicle (PBS; 1 mL/kg) treated animals served as a control group (Figure 6E). The body temperature was registered by a rectal thermometer and was controlled by the RightTemp module (Figure 6F). The body temperature at 0 min was 36.5 ± 0.7 °C and was kept at a stable level during the whole experiment.

### 4.5. Experimental Procedure Performed with Both Methods Separately

Thirty male Wistar rats (12–15 weeks old, weighing 234.7 ± 31.1 g) were randomly divided into 6 groups, anesthetized by an intraperitoneal injection of pentobarbital (45 mg/kg), and placed in a supine position on a heated operation table. Supplemental pentobarbital (5 mg/kg) was administered as needed, with a delay of at least 10 min before any experimental measurements were made. The drugs were administered after the stabilization of measured parameters. Adrenaline (5 µg/kg, 1 mL/kg) and nitroglycerin (100 µg/kg, 1 mL/kg) were administered into the right femoral vein. Vehicle (PBS; 1 mL/kg) treated animals served as a control group. HR, blood perfusion of rat’s paw, blood oxygen saturation, respiratory rate, average and peak exhaled CO_2_, and body temperature were measured using the PhysioSuite Physiological Monitoring Modular System (Kent Scientific Corporation, Torrington, CT, USA) for 60 min after drugs administration. Systolic, diastolic, and mean BP were measured according to the methods described above with pressure transducer (Plugsys, Transonics System Inc., Ithaca, NY, USA). The body temperature was controlled by the RightTemp module.

### 4.6. Statistical Analysis

In the study, ‘n’ refers to the number of animals in each experimental group. We chose a minimal number of animals to detect the differences between each group based on our experience as well as others’ experience using similar procedures. With the aid of GraphPad Prism 8 (GraphPad Software, La Jolla, CA, USA), groups were compared by 1-way ANOVA or the Kruskal–Wallis test. Post hoc group comparisons were performed by Dunnett test or the Dunn method, respectively. In case of comparing only two groups of data we used unpaired Student’s *t*-test or Mann–Whitney test. Usage of parametric or nonparametric tests were determined whenever deviation either from normality of residuals or from homogeneity of variances was observed according to the Shapiro–Wilk statistic or F-test, respectively. The Spearman correlation coefficient was used to assess the correlation of blood perfusion of paw vessels with mean BP. Data are mean ± SD or median with range according to distribution. The results were graphically presented using BioRender (Toronto, ON, Canada) or GraphPad Prism 8 (GraphPad Software, La Jolla, CA, USA). *p* values < 0.05 were considered significant.

## 5. Conclusions

We have demonstrated the reliability, accuracy, and sensitivity of the developed method based on the known effects of adrenaline and nitroglycerin clearly described in the literature. We achieved similar results using both devices combined or separately, which proves a new method does not interfere with the measurement of cardiovascular and respiratory parameters simultaneously. We are aware that we cannot examine all parameters recommended by ICH S7A in our method but achieved results which allow us to draw general conclusions about its safety and may predict toxicity in future clinical studies. Observation of nine vital parameters in one rat at the same time allows to significantly reduce the number of animals used in the study. Our method using direct and indirect techniques is fast, conducted without stress and does not require long acclimatization of animals. We believe this method is appropriate for early evaluation of the safe pharmacology and toxicology parameters of short-acting drug candidates administrated intravenously.

## Figures and Tables

**Table 1:** Change of blood pressure in anaesthetized rats after administration of drugs.

	Adrenaline Dose			Nitroglycerin Dose		
T (min)	Low	Medium	High	Low	Medium	High
Δ Systolic blood pressure (mmHg)						
0.5	4.3 (0.9–8.3)	22.2 (10.9–26.4) ^b^	63.9 (39.9–67.0) ^c^	−10.6 (−17.6–−5.7)	−36.7 (−44.0–−31.4) ^b^	−62.5 (−74.0–−56.8) ^c^
1	−1.7 (−3.7–1.8)	8.9 (4.4–13.5)	29.1 (21.2–57.8) ^b^	−6.8 (−10.0–−2.3)	−28.2 (−40.5–10.3) ^a^	−46.5 (−64.0–−37.6) ^c^
2	−0.9 (−2.0–2.2)	−5.3 (−6.1–−0.2)	0.0 (−8.4–6.8)	1.1 (−0.1–4.4)	−11.1 (−23.2– −3.4)	−27.4 (−49.8–−9.4) ^a^
3	−1.3 (−3.9–0.9)	−1.6 (−6.2–0.3)	−6.4 (−13.4–−5.0) ^c^	3.7 (1.4–7.4)	−2.8 (−7.0–1.5)	−15.6 (−44.2–1.4)
10	−3.3 (−5.2–3.1)	6.4 (−0.2–10.3)	−3.0 (−9.2–4.5)	13.7 (8.8–17.0) ^a^	8.4 (2.9–13.9)	4.8 (−2.6–15.1)
30	−10.9 (−21.4–3.6)	10.3 (−5.9–24.4)	0.6 (−7.3–13.5)	10.4 ± 2.5	4.3 ± 6.8	4.9 ± 11.7
60	−21.5 (−24.9–9.5)	10.1 (−16.9–24.0)	−2.9 (−10.9–13.5)	6.8 ± 4.6	−2.6 ± 6.1	2.9 ± 11.5
Δ Diastolic blood pressure (mmHg)						
0.5	4.0 (0.9–7.6)	13.1 (7.6–23.4) ^a^	55.5 (33.2–65.9) ^c^	−9.3 (−15.5–−6.1)	−34.4 (−41.9–−30.7) ^b^	−61.5 (−73.0–−53.8) ^c^
1	−0.8 (−4.0–0.8)	7.3 (0.2–10.9)	25.2 (17.9–50.6) ^b^	−6.4 (−9.3–−1.9)	−27.1 (−40.1–−10.9) ^a^	−46.5 (−66.0–−42.7) ^c^
2	−0.6 ± 2.0	−4.8 ± 3.8	−2.8 ± 7.4	1.8 (1.1–4.6)	−11.1 (−19.3–−2.4)	−25.2 (−48.6–−11.1) ^a^
3	−0.2 (−4.0–1.5)	−3.5 (−7.1–−0.2)	−9.2 (−14.0–−6.2) ^c^	4.6 (2.3–7.8)	−1.4 (−6.5–2.8)	−12.9 (−42.2–2.2)
10	−3.0 (−5.5–4.7)	7.9 (0.5–9.9)	−2.2 (−8.2–6.0)	15.7 (9.7–19.1) ^a^	10.3 (3.4–15.0)	6.2 (−7.6–17.0)
30	−10.7 (−21.0–4.8)	9.9 (−5.4–23.3)	1.3 (−6.2–13.5)	12.7 ± 2.3	5.1 ± 8.0	5.7 ± 12.9
60	−17.8 (−24.6–9.5)	10.7 (−15.1–22.8)	−3.2 (−12.0–10.6)	9.2 ± 3.0	−0.5 ± 7.1	3.9 ± 11.4
Δ Mean blood pressure (mmHg)						
0.5	4.3 (1.1–8.0)	15.5 (13.5–24.9) ^b^	56.4 (36.8–66.5) ^c^	−9.8 (−16.6–−5.9)	−35.6 (−42.2–−31.4) ^b^	−62.0 (−73.5–−55.4) ^c^
1	−1.4 (−3.9–0.1)	8.9 (−0.1–12.3)	27.2 (19.6–52.1) ^b^	−6.6 (−10.1–−1.9)	−27.2 (−39.8–−11.0) ^a^	−45.3 (−63.5–−37.6) ^c^
2	−0.8 ± 1.9	−4.3 ± 3.4	−2.6 ± 6.7	1.4 (0.7–3.9)	−11.4 (−21.3– −3.0)	−26.7 (−49.1–−10.2) ^a^
3	−1.0 (−4.2–1.1)	−2.6 (−6.7–1.1)	−8.7 (−17.4–−5.8) ^c^	4.1 (1.8–7.2)	−2.5 (−6.5–2.0)	−14.5 (−43.1–1.8)
10	−2.9 (−5.6–3.8)	7.7 (0.9–10.1)	−3.1 (−10.0–5.1)	14.3 (9.4–17.5) ^a^	8.9 (2.6–14.3)	5.6 (−4.2–15.7)
30	−10.8 (−21.0–4.0)	10.1 (−5.7–23.9)	1.0 (−6.6–13.6)	11.2 ± 2.4	4.6 ± 7.4	5.3 ± 12.2
60	−19.6 (−24.7–9.1)	10.4 (−16.0–23.4)	−3.1 (−11.0–13.3)	7.9 ± 3.8	−1.7 ± 6.5	3.1 ± 11.4

Data are median with range or mean ± SD (n = 5–8). Δ indicates the change in relation to the value at the 0 min of the experiment in the test group; T, time and min, minute. ^a^ *p* < 0.05, ^b^ *p* < 0.01, ^c^ *p* < 0.001 vs. vehicle with Dunnett test in 1-way ANOVA or the Dunn method in Kruskal–Wallis.

**Table 2:** Change of blood perfusion, heart rate, and blood oxygen saturation in anaesthetized rats after administration of drugs.

	Adrenaline Dose			Nitroglycerin Dose		
T (min)	Low	Medium	High	Low	Medium	High
Δ Blood perfusion (%)						
0.5	0.0 (−0.01–0.02)	0.01 (−0.01–0.03)	0.08 (0.0–0.12) ^a^	0.01 (0.0–0.03)	0.06 (0.02–0.09) ^a^	0.19 (0.0–0.29) ^b^
1	0.0 (−0.01–0.02)	0.01 (0.0–0.04) ^a^	0.03 (0.01–0.1) ^b^	0.01 (0.0–0.01)	0.05 (0.02–0.09) ^a^	0.19 (0.17–0.21) ^c^
2	0.0 (0.0–0.03)	−0.01 (−0.04–0.02)	−0.03 (−0.04–0.01)	0.01 (−0.01–0.01)	0.02 (0.0–0.03) ^a^	0.03 (0.01–0.04) ^b^
3	0.03 (0.01–0.03)	−0.01 (−0.03–0.02)	−0.02 (−0.04–0.01)	0.0 (−0.01–0.01)	0.01 (0.0–0.02)	0.0 (0.0–0.02)
10	0.01 (−0.01–0.05) ^a^	0.0 (−0.01–0.03)	−0.01 (−0.01–0.0)	0.01 (−0.01–0.02)	0.03 (0.0–0.04) ^b^	0.01 (−0.01–0.06)
30	−0.01 (−0.01–0.02)	0.0 (−0.05–0.01)	0.0 (−0.01–0.01) ^a^	0.0 (−0.01–0.01)	0.02 (0.0–0.03) ^b^	−0.01 (−0.03–0.08)
60	−0.02 (−0.03–0.0)	−0.01 (−0.06–−0.01)	−0.01 (−0.01–0.0) ^a^	−0.01 (−0.03–0.0)	0.0 (−0.02–0.01) ^a^	−0.01 (−0.03–0.07)
Δ Heart rate (bpm)						
0.5	−2.0 (−4.0–0.0)	11.0 (3.0–21.0)	43.0 (35.0–47.0) ^b^	2.0 (−33.0–5.0)	11.0 (−4.0–16.0)	12.0 (0.0–27.0)
1	−6.0 (−16.0–4.0)	21.0 (16.0–31.0)	69.0 (−61.0–102.0) ^b^	−2.0 (−10.0–0.0)	9.0 (6.0–33.0) ^a^	12.0 (3.0–30.0) ^a^
2	−5.0 (−17.0–3.0)	22.0 (17.0–34.0)	57.0 (43.0–82.0) ^b^	−5.0 (−8.0–3.0)	15.0 (8.0–42.0) ^b^	13.0 (−2.0–30.0)
3	−6.0 (−16.0–0.0)	25.0 (10.0–36.0)	35.0 (18.0–61.0) ^a^	−8.0 (−10.0–9.0)	38.0 (26.0–50.0) ^a^	18.0 (1.0–30.0)
10	−12.0 (−23.0–−3.0) ^a^	9.0 (−13.0–16.0)	2.0 (−9.0–33.0)	9.0 (2.0–30.0)	20.0 (16.0–29.0)	32.0 (8.0–43.0) ^a^
30	−25.0 (−34.0–−9.0)	19.0 (0.0–36.0)	8.0 (−42.0–50.0)	15.0 (7.0–33.0)	22.0 (18.0–33.0) ^b^	24.0 (−11.0–50.0)
60	−38.0 (−48.0–−3.0)	15.0 (5.0–53.0)	43.0 (−56.0–55.0)	8.0 (−2.0–40.0)	1.0 (1.0–13.0)	26.0 (−22.0–35.0)
Δ Blood oxygen saturation (%)						
0.5	−14.8 ± 19.4	−9.4 ± 13.2	1.2 ± 11.1	0.0 (−34.0–10.0)	8.0 (−6.0–15.0)	7.0 (−21.0–12.0)
1	−0.6 ± 6.3	−0.2 ± 6.1	−1.0 ± 10.9	−3.4 ± 6.5	−4.8 ± 3.0	−4.8 ± 7.4
2	−4.0 (−9.0–5.0)	0.0 (−16.0–2.0)	−13.0 (−44.0–0.0) ^a^	−6.0 (−13.0–−2.0) ^a^	1.0 (−3.0–2.0)	1.0 (−3.0–4.0)
3	−4.0 ± 4.5	−6.0 ± 7.7	−14.2 ± 18.4^a^	−7.0 (−11.0–−3.0)	3.0 (0.0–3.0)	4.0 (−9.0–5.0)
10	1.0 (−2.0–4.0)	0.0 (−6.0–18.0)	1.0 (−13.0–3.0)	−6.0 (−8.0–3.0)	−3.0 (−5.0–6.0)	1.0 (−8.0–7.0)
30	−6.0 (−11.0–−4.0)	−11.0 (−19.0–4.0)	−23.0 (−42.0–0.0) ^a^	−5.0 ± 5.8	−1.8 ± 3.3	−3.6 ± 4.7
60	−14.0 (−25.0–−11.0) ^a^	−8.0 (−23.0–3.0)	−12.0 (−13.0–−8.0)	−4.2 ± 3.1	−3.4 ± 2.2	−6.2 ± 6.8

Data are median with range or mean ± SD (n = 5–8). Δ indicates the change in relation to the value at the 0 min of the experiment in the test group; T, time and min, minute. ^a^ *p* < 0.05, ^b^ *p* < 0.01, ^c^ *p* < 0.001 vs. vehicle with Dunnett test in 1-way ANOVA or the Dunn method in Kruskal–Wallis.

**Table 3:** Change of respiratory rate and peak CO_2_ in anaesthetized rats after administration of drugs.

	Adrenaline Dose			Nitroglycerin Dose		
T (min)	Low	Medium	High	Low	Medium	High
Δ Respiratory rate (bpm)						
0.5	2.2 ± 2.0	2.2 ± 6.7	−0.4 ± 7.9	0.8 ± 5.2	5.0 ± 4.5	3.0 ± 8.6
1	6 (2–23) ^b^	2 (1–4)	4 (2–17) ^a^	−1.0 (−2.0–0.0)	0.0 (−2.0–5.0)	5.0 (2.0–8.0)
2	1.2 ± 1.8	3.2 ± 4.8	5.4 ± 4.5	0.0 (−1.0–2.0)	1.0 (−2.0–7.0)	7.0 (1.0–7.0)
3	2.4 ± 2.2	3.2 ± 3.7	6.4 ± 6.2	2.0 (−3.0–5.0)	4.0 (1.0–5.0)	6.0 (3.0–10.0) ^b^
10	0.6 ± 2.7	1.2 ± 3.0	0.0 ± 6.8	13.0 (5.0–20.0) ^a^	14.0 (7.0–18.0) ^a^	13.0 (9.0–17.0) ^b^
30	−1.8 ± 3.7	3.2 ± 2.8	0.4 ± 7.2	21.0 (14.0–34.0) ^b^	17.0 (8.0–19.0)	17.0 (1.0–21.0) ^a^
60	1.0 ± 3.5	3.4 ± 3.5	4.6 ± 2.8	26.0 (10.0–31.0) ^b^	21.0 (10.0–24.0)	19.0 (7.0–30.0)
Δ Peak CO_2_ (%)						
0.5	0.1 ± 0.6	0.0 ± 0.1	−0.1 ± 0.2	−0.1 ± 0.2	−0.4 ± 0.4	−0.1 ± 0.1
1	0.1 ± 0.2	0.1 ± 0.1	0.1 ± 0.1	0.0 (−0.1–0.1)	−0.1 (−0.2–0.0)	−0.1 (−0.4–−0.1) ^a^
2	0.1 ± 0.2	0.1 ± 0.1	0.2 ± 0.1	0.0 ± 0.1	−0.1 ± 0.1	−0.1 ± 0.2
3	0.1 ± 0.1	0.0 ± 0.1	0.1 ± 0.1	−0.1 ± 0.1	0.0 ± 0.0	0.0 ± 0.2
10	0.2 ± 0.1	0.0 ± 0.1	0.0 ± 0.1	−0.2 (−0.3–−0.1) ^a^	0.0 (−0.3–0.1)	0.1 (−0.1–0.1)
30	0.2 (0.1–0.5)	−0.1 (−0.3–−0.1) ^a^	−0.1 (−0.2–0.2)	−0.3 (−0.4–−0.2) ^c^	−0.1 (−0.3–0.0)	−0.1 (−0.4–0.1)
60	0.1 (−0.1–0.3)	−0.1 (−0.2–0.0)	−0.2 (−0.3–−0.1)	−0.4 (−0.4–−0.3 ) ^b^	−0.2 (−0.2–−0.1)	−0.2 (−0.2–−0.1)

Data are median with range or mean ± SD (n = 5–8). Δ indicates the change in relation to the value at the 0 min of the experiment in the test group; T, time and min, minute. ^a^ *p* < 0.05, ^b^ *p* < 0.01, ^c^ *p* < 0.001 vs. vehicle with Dunnett test in 1-way ANOVA or the Dunn method in Kruskal–Wallis.

## Data Availability

Data is contained within the article and supplementary files.

## Supplementary Materials

The following are available online at https://www.mdpi.com/article/10.3390/ph14121223/s1, Table S1: The values of tested parameters at 0 min of the experiment carried out with the use of two methods simultaneously, Table S2: Change of blood pressure in anaesthetized rats after administration of drugs measured by Plugsys (Transonics System Inc.), Table S3: Change of blood perfusion, heart rate and blood oxygen saturation in anaesthetized rats after administration of drugs measured by the PhysioSuite Physiological Monitoring Modular System (Kent Scientific Corporation), Table S4: Change of respiratory rate and peak CO_2_ in anaesthetized rats after administration of drugs measured by the PhysioSuite Physio-logical Monitoring Modular System (Kent Scientific Corporation), Table S5: The values of tested parameters at 0 min of the experiment carried out with the use of two methods separately.
