# Supplementary material for: Monitoring of Cardiorespiratory Parameters in Rats—Validation Based on Pharmacological Stimulation
Source: Pharmaceuticals (Basel). 2021 Nov 25;14(12):1223. doi: 10.3390/ph14121223 (PMC8705495; doi:10.3390/ph14121223)
Supplement: Supplementary file 1 [file pharmaceuticals-14-01223-s001.zip › pharmaceuticals-1445323-supplementary.pdf]

pharmaceuticals

Supplementary Materials

Monitoring of Cardiorespiratory Parameters in Rats – Validation Based on Pharmacological Stimulation

Joanna Miklosz [1*], Bartlomiej Kalaska [1], Stanislaw Zajaczkowski [2], Dariusz Pawlak [1] and Andrzej Mogielnicki [1]

[1] Department of Pharmacodynamics, Medical University of Bialystok, 15-089 Bialystok, Poland; bartlomiej.kalaska@umb.edu.pl; dariusz.pawlak@umb.edu.pl; andrzej.mogielnicki@umb.edu.pl
[2] Department of Physiology, Medical University of Gdansk, 80-211 Gdansk, Poland; stanislaw.zajaczkowski@gumed.edu.pl
* Correspondence: joanmiklosz@gmail.com; Tel.: +48-85-748-5231

Table S1. The values of tested parameters at 0 minute of the experiment carried out with the use of two methods simultaneously.

		Adrenaline dose			Nitroglycerin dose		
**Parameters**	**Vehicle**	**low**	**medium**	**high**	**low**	**medium**	**high**
**Systolic blood pressure (bpm)**	126.2 (110.5-141.1)	125.3 (115.7-148.6)	120.2 (90.3-148.3)	119.5 (102.2-127.3)	114.2 (108.0-120.3)	127.4 (105.1-141.2)	118.8 (112.2-127.1)
**Diastolic blood pressure (bpm)**	118.4 (110.0-135.2)	121.1 (110.8-139.9)	115.6 (86.8-140.9)	113.7 (98.6-122.9)	107.7 (100.5-113.2)	120.5 (99.0-134.2)	114.7 (108.7-119.5)
**Mean blood pressure (bpm)**	122.3 (110.2-138.0)	123.4 (113.6-144.3)	117.7 (88.6-144.6)	118.6 (100.3-125.2)	111.3 (104.4-116.8)	124.1 (101.9-137.9)	116.8 (110.9-123.8)
**Blood perfusion (%)**	0.08 (0.04-0.19)	0.07 (0.06-0.11)	0.05 (0.04-0.09)	0.05 (0.02-0.08)	0.04 (0.02-0.08)	0.05 (0.03-0.06)	0.05 (0.04-0.06)
**Heart rate (bpm)**	439 (382-487)	405 (379-473)	411 (340-457)	374 (347-391)	395 (379-410)	417 (367-457)	379 (321-412)
**Blood oxygen saturation (%)**	85 (72-94)	82 (62-84)	79 (64-81)	80 (69-85)	83 (74-89)	82 (78-88)	86 (82-88)
**Respiratory rate (bpm)**	72 (42-88)	65 (59-77)	60 (51-76)	64 (56-71)	52 (41-65)	66 (50-74)	56 (45-65)
**Peak CO_2 (%)**	4.2 (3.6-4.3)	4.3 (3.7-4.7)	3.8 (3.5-4.2)	3.9 (3.7-4.0)	4.3 (3.9-5.2)	4.1 (3.7-4.5)	4.1 (3.8-4.7)

Data are median with range (n = 5–8).

Table S2. Change of blood pressure in anaesthetized rats after administration of drugs measured by Plugsys (Transonics System) separately.

T (min)	Adrenaline 5 µg/kg	Nitroglycerin 10 µg/kg
**Δ Systolic blood pressure (mmHg)**		
**0.5**	**38.7±14.3[c]**	**-57.7±7.6[c]**
**1**	**31.7 (3.9–49.3)[a]**	**-46.9 (-57.7– -21.5)[b]**
**2**	-7.8±10.5	**-25.9 (-47.3– -6.9)[b]**
**3**	-6.6±6.6	-13.6±13.8
**10**	4.1±8.3	1.2±10.4
**30**	3.9±8.6	-1.4±10.0
**60**	3.2±8.6	-4.6±7.1
**Δ Diastolic blood pressure (mmHg)**		
**0.5**	**31.1 (12.0–60.1)[b]**	**-56.7±6.6[c]**
**1**	**28.20 (2.6–49.2)[a]**	**-45.5 (-52.7– -23.1)[b]**
**2**	-7.5±9.9	**-21.3±13.9[b]**
**3**	-6.6±6.0	-11.8±12.1
**10**	4.4±7.1	3.2±7.1
**30**	4.9±8.3	-0.2±6.2
**60**	3.3 (-6.0– 17.4)	-2.6 (-10.0– -0.7)
**Δ Mean blood pressure (mmHg)**		
**0.5**	**36.6±15.9[b]**	**-57.2±6.2[c]**
**1**	**30.2 (3.3–49.2)[a]**	**-46.6 (-55.4– -22.3)[b]**
**2**	-7.7±10.2	**-23.6 (-44.8– -8.4)[b]**
**3**	-6.6±6.3	-12.6±13.0
**10**	4.1±7.9	2.2±8.8
**30**	4.3±8.4	-0.8±8.2
**60**	3.7±8.5	-4.0±5.3

Data are median with range or mean ± SD (n = 5). Δ indicates the change in relation to the value at the 0 minute of the experiment in the test group; T, time and min, minute.
[a]P<0.05, [b]P<0.01, [c]P<0.001 vs vehicle with Mann–Whitney test or the unpaired Student's t-test.

Table S3. Change of blood perfusion, heart rate and blood oxygen saturation in anaesthetized rats after administration of drugs measured by the PhysioSuite Physiological Monitoring Modular System (Kent Scientific Corporation) separately.

T (min)	Adrenaline 5 µg/kg	Nitroglycerin 10 µg/kg
Δ Blood perfusion (%)		
0.5	**0.12 (0.09–0.14)[b]**	**0.19 (0.14–0.25)[b]**
1	0.02 (-0.04–0.08)	**0.07 (0.01–0.1)[a]**
2	-0.04±0.04	0.01±0.03
3	-0.03±0.04	0.01±0.01
10	0.01±0.02	0.03±0.04
30	0.01±0.02	0.03±0.04
60	0.01±0.02	0.03±0.04
Δ Heart rate (bpm)		
0.5	**48.0 (47.0–124.0)[b]**	-9.0 (-14.0–0.0)
1	**57.0 (40.0–107.0)[b]**	-10.4±13.2
2	**41.2±17.3[c]**	-7.6±16.9
3	**32.0 (20.0–56.0)[b]**	-8.0 (-21.0–7.0)
10	10.0±24.7	-6.0 (-17.0–49.0)
30	8.0 (-32.0–36.0)	5.8±22.3
60	8.0 (-35.0–36.0)	4.6±21.6
Δ Blood oxygen saturation (%)		
0.5	-5.0 (-27.0–0.0)	0.0 (-27.0–6.0)
1	-2.0 (-37.0–0.0)	-4.0 (-37.0–5.0)
2	**-12.8±4.4[a]**	-7.6±5.7
3	-7.6±9.6	-3.2±7.4
10	3.0 (-7.0–29.0)	8.0 (-7.0–10.0)
30	1.8±11.7	-3.0±6.7
60	1.6±11.8	-4.8±6.1

Data are median with range or mean ± SD (n = 5). Δ indicates the change in relation to the value at the 0 minute of the experiment in

the test group; T, time and min, minute.
[a]P<0.05, [b]P<0.01, [c]P<0.001 vs vehicle with Mann–Whitney test or the unpaired Student's t-test.

Table S4. Change of respiratory rate and peak CO_2 in anaesthetized rats after administration of drugs measured by the PhysioSuite Physiological Monitoring Modular System (Kent Scientific Corporation) separately.

T (min)	Adrenaline 5 µg/kg	Nitroglycerin 10 µg/kg
Δ Respiratory rate (bpm)		
0.5	-3.0 (-30.0–5.0)	2.6±2.5
1	-4.2±4.1	0.0±4.2
2	0.2±6.4	1.8±3.1
3	3.2±4.9	3.8±3.1
10	0.0 (0.0–7.0)	8.0 (-1.0–20.0)
30	-1.0 (-1.0–7.0)	10.0 (0.0–20.0)
60	-1.0 (-2.0–8.0)	9.0 (1.0–20.0)
Δ Peak CO_2 (%)		
0.5	-0.04 (-1.89– -0.02)	-0.11 (-0.30– -0.08)
1	0.04 (-0.21–0.33)	**-0.11±0.07**[a]
2	0.09 (-0.01–0.77)	-0.06 (-0.12–0.05)
3	0.02 (-0.76–0.53)	-0.02±0.05
10	-0.05±0.23	-0.04±0.18
30	-0.04±0.18	-0.05±0.16
60	-0.05±0.18	-0.05±0.17

Data are median with range or mean ± SD (n = 5). Δ indicates the change in relation to the value at the 0 minute of the experiment in the test group; T, time and min, minute.
[a]$P<0.05$, [b]$P<0.01$, [c]$P<0.001$ vs vehicle with Mann–Whitney test or the unpaired Student's t-test.

Table S5. The values of tested parameters at 0 minute of the experiment carried out with the use of two methods separately.

Parameters	Vehicle	Adrenaline	Nitroglycerin
Systolic blood pressure (bpm)	111.3 (82.6-142.9)	101.8 (90.3-135.8)	125.0 (105.5-134.5)
Diastolic blood pressure (bpm)	106.6 (77.3.0-138.9)	95.4 (89.2-129.7)	118.5 (99.7-127.2)
Mean blood pressure (bpm)	109.0 (80.3-141.0)	100.1 (89.8-132.9)	121.9 (102.7-130.6)
Blood perfusion (%)	0.05 (0.04-0.11)	0.08 (0.05-0.14)	0.07 (0.03-0.08)
Heart rate (bpm)	431 (366-445)	415 (351-443)	378 (338-484)
Blood oxygen saturation (%)	88 (80-96)	86 (70-99)	87 (86-99)
Respiratory rate (bpm)	69 (55-73)	69 (59-81)	63 (41-75)
Peak CO_2 (%)	2.3 (1.7-2.8)	2.3 (1.8-3.1)	2.8 (2.0-2.9)

Data are median with range (n = 5).
